# Evidence for Endogenous Opioid Dependence Related to Latent Sensitization in a Rat Model of Chronic Inflammatory Pain

**DOI:** 10.3390/ijms24032812

**Published:** 2023-02-01

**Authors:** Julio César Morales-Medina, Nicola Pugliese, Alessandro Di Cerbo, Claudia Zizzadoro, Tommaso Iannitti

**Affiliations:** 1 Centro de Investigación en Reproducción Animal, CINVESTAV-Universidad Autónoma de Tlaxcala, AP 62, Tlaxcala 90000, Mexico; 2 Department of Veterinary Medicine, University of Bari, 70010 Valenzano, Italy; 3 School of Biosciences and Veterinary Medicine, University of Camerino, 62024 Matelica, Italy; 4 Section of Experimental Medicine, Department of Medical Sciences, University of Ferrara, Via Fossato di Mortara 70, 44121 Ferrara, Italy

**Keywords:** rodent models, complete Freund’s adjuvant, peripheral inflammation, endogenous opioids, species differences, nociceptive reinstatement

## Abstract

Studies performed in a mouse model of chronic inflammatory pain induced by intraplantar injection of complete Freund’s adjuvant (CFA) have shown that constitutive activation of the endogenous opioid signaling, besides serving as a mechanism of endogenous analgesia that tonically represses pain sensitization, also generates a state of endogenous opioid dependence. Since species-related differences concerning pain biology and addictive behaviors occur between mice and rats, the present study explored whether the coexistence of endogenous opioid analgesia and endogenous opioid dependence also characterizes a homologous rat model. To this aim, CFA-injured Wistar rats were treated with either 3 mg/kg or 10 mg/kg of the opioid receptor inverse agonist naltrexone (NTX) during the pain remission phase and monitored for 60 min for possible withdrawal behaviors. At 3 mg/kg, NTX, besides inducing the reinstatement of mechanical allodynia, also caused a distinct appearance of ptosis, with slight but nonsignificant changes to the occurrence of teeth chatters and rearing. On the other hand, 10 mg/kg of NTX failed to unmask pain sensitization and induced significantly lower levels of ptosis than 3 mg/kg. Such an NTX-related response pattern observed in the rat CFA model seems to differ substantially from the pattern previously described in the mouse CFA model. This supports the knowledge that mice and rats are not identical in terms of pharmacological response and stresses the importance of choosing the appropriate species for preclinical pain research purposes depending on the scientific question being asked.

## 1. Introduction

Pain prevalence ranges from 38.4 to 49.6% in developed countries and from 24.1 to 60.4% in developing countries [1], while chronic pain is a major health problem worldwide [2]. Its development involves complex and dramatic changes to the neural pain pathways from the peripheral nociceptors to the cerebral cortex, leading to a persistent state of hypersensitivity [2,3,4,5].

Therefore, comprehensive identification of these changes and underlying molecular mechanisms, as well as of the factors that increase individual susceptibility, is pivotal to improving medical strategies to minimize or eliminate the potential risk of transition from acute (protective) to chronic (pathological) pain in clinical practice.

Rodent models of persistent pain are essential experimental tools for understanding chronic pain and assessing the analgesic efficacy of candidate compounds in the drug discovery and development process [6]. Among the numerous models developed over many decades to recreate the various types of chronic pain conditions affecting human beings [4], the complete Freund’s adjuvant (CFA) model [7,8] has proven to be particularly suitable for studying the pathogenesis of chronic inflammatory pain in vivo [9]. Indeed, the peripheral inflammatory response induced by the injection of this chemical agent into the hind paw of rodents is robust and persistent enough to result in a pronounced sensitization and consequent hyperreactivity that involve not only the nociceptors at the site of the chemical injury but also the nociceptive signaling pathways of the central nervous system (CNS) [10]. Central sensitization can be defined as an abnormal responsiveness state or increased gain in the pain system [11]. Central pain sensitization associated with CFA-induced inflammatory injury does not end upon the resolution of tissue injury and disappearance of overt signs of hyperalgesia but persists for several months in a silent form termed latent sensitization [9,12,13,14]. In large part, such a state of remission, defined as a period with no mechanical allodynia, is initiated and maintained by the constitutive activity of a population of μ-opioid receptors (MORs) engaged in the CNS to suppress the activity of sensitized pronociceptive neurons tonically. Consistently, by the administration of a centrally acting MOR inverse agonist such as naltrexone (NTX) during the remission phase, it is possible to transiently unmask the central sensitization and reinstate neuronal nociceptive activity in the CNS with signs of pain hypersensitivity at the injury site [9,12,13,14]. 

Based on these characteristics, the rodent model of CFA-induced chronic inflammatory pain and latent sensitization is considered able to reproduce the episodic nature and vulnerability to stressors that typically accompany chronic pain states in clinical patients (with exposure to excessive stress being a known cause of the loss of opioid inhibitory control on central nociceptive circuitry) [9].

Studies performed in CFA-injured mice [12,13,14] have revealed that, along with long-term endogenous analgesia, the induction of MOR constitutive activity (MOR_CA_) in the brain also generates a state of endogenous opioid physical dependence through mechanisms of compensatory neuroadaptations that likely resemble those involved in the development of addiction following repeated treatment with exogenous opiates [12]. In support of this, it was found that NTX administration under conditions of latent sensitization also precipitated opioid withdrawal behaviors in mice. This finding suggested that the MOR_CA_ induced within the CNS by the peripheral inflammatory injury may not only act as an endogenous mechanism to prevent the transition from acute to chronic pain but also paradoxically promote at the same time the long-term persistence of central pain sensitization, creating a lasting predisposition to pain relapse [12]. 

Appropriately targeting the opposing homeostatic interactions between the endogenous antinociceptive opioid receptor signaling and the latent pronociceptive signaling in the CNS may thus offer new options to prevent or manage chronic pain states [14].

Besides mice, rats also have been used to develop the CFA model of chronic inflammatory pain [7,8,9]. However, whether the increased endogenous opioid tone responsible for long-term endogenous analgesia induces endogenous physical dependence in this rodent species is still unknown. Considering the well-documented existence of differences in pain biology and addictive behaviors of rats and mice [15,16] and given the potentially important impact that such interspecies differences may have on the translational validity of the two rodent models [15,16], the present study was designed to explore the possible occurrence of endogenous opioid dependence in CFA-injured rats under conditions of MOR_CA_-dependent latent sensitization. The experimental work was conducted using Wistar rats because of the experience gained with this strain in our laboratory during previous pain studies [17,18,19,20]. However, current knowledge regarding latent pain sensitization induced by chronic peripheral inflammation in rats is exclusively referred to the Sprague–Dawley strain [9,18,21], and differences relevant to the pain biology [16] and behavioral responses [19] have been reported to also occur between these two rat strains. Therefore, the design of our study also included control experiments using sham-injured rats as comparators to verify that, in the Wistar strain too, the intraplantar injection of CFA is an effective and specific stimulus for inducing the basic phases of latent sensitization and that possible pronociceptive and withdrawal precipitating effects of NTX is dependent on the presence of a condition of latent sensitization.

## 2. Results

### 2.1. Time-Course of CFA-Induced Inflammation and Pain in Male Wistar Rats

**Figure 1:**
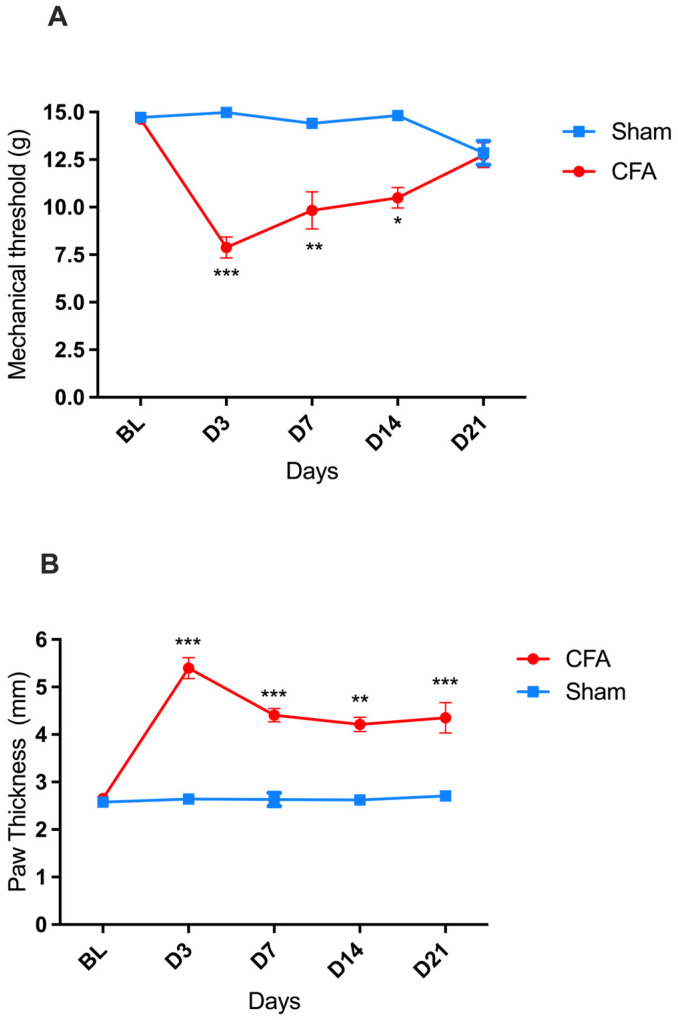
CFA-induced pain (mechanical allodynia) and inflammation (paw edema) in male Wistar rats. (**A**) Progression of mechanical hypersensitivity, measured as mechanical withdrawal response thresholds to von Frey filaments, after intraplantar injection of CFA (●; *n* = 17) or sham paw injury with saline (■; *n* = 7). (**B**) Progression of paw edema, measured as the diameter of the left hind paw, after intraplantar injection of CFA (●; *n* = 17) or sham paw injury with saline (■; *n* = 7). * *p* < 0.05, ** *p* < 0.01, *** *p* < 0.001. Each point represents mean ± SEM. See main text for abbreviations.

A significant decrease in the paw mechanical withdrawal threshold (indicative of mechanical allodynia) was recorded in rats on days three, seven, and fourteen after the intraplantar injection of CFA compared to baseline (BL), with a greater decrease observed on day three (D3). By D21, the mechanical withdrawal threshold of the CFA-injured rats returned to BL values [two-way ANOVA, time F(4,88) = 6.360 *** *p* < 0.001, treatment F(1,22) = 33.19 *** *p* < 0.001, and interaction F(4,88) = 10.22 *** *p* < 0.001] (Figure 1A). In parallel, a significant increase in the thickness of the injected paw (indicative of paw edema) was detected, reaching its maximum on D3 and then followed by a gradual reduction in amplitude down to its lowest (but still significant) value on D21 [two-way ANOVA, time F(4,88) = 11.83 *p* < 0.001, treatment F(1,22) = 90.41 *** *p* < 0.001, and interaction F(4,88) = 10.59 *** *p* < 0.001] (Figure 1B).

In the sham-injured rats, no change in the mechanical threshold or paw thickness was recorded during the 21 days observation period after the saline injection.

### 2.2. Effect of NTX Administration on the Paw Mechanical Withdrawal Threshold of Male Wistar Rats at 21 Days Post-CFA Administration (CFA-21d)

**Figure 2:**
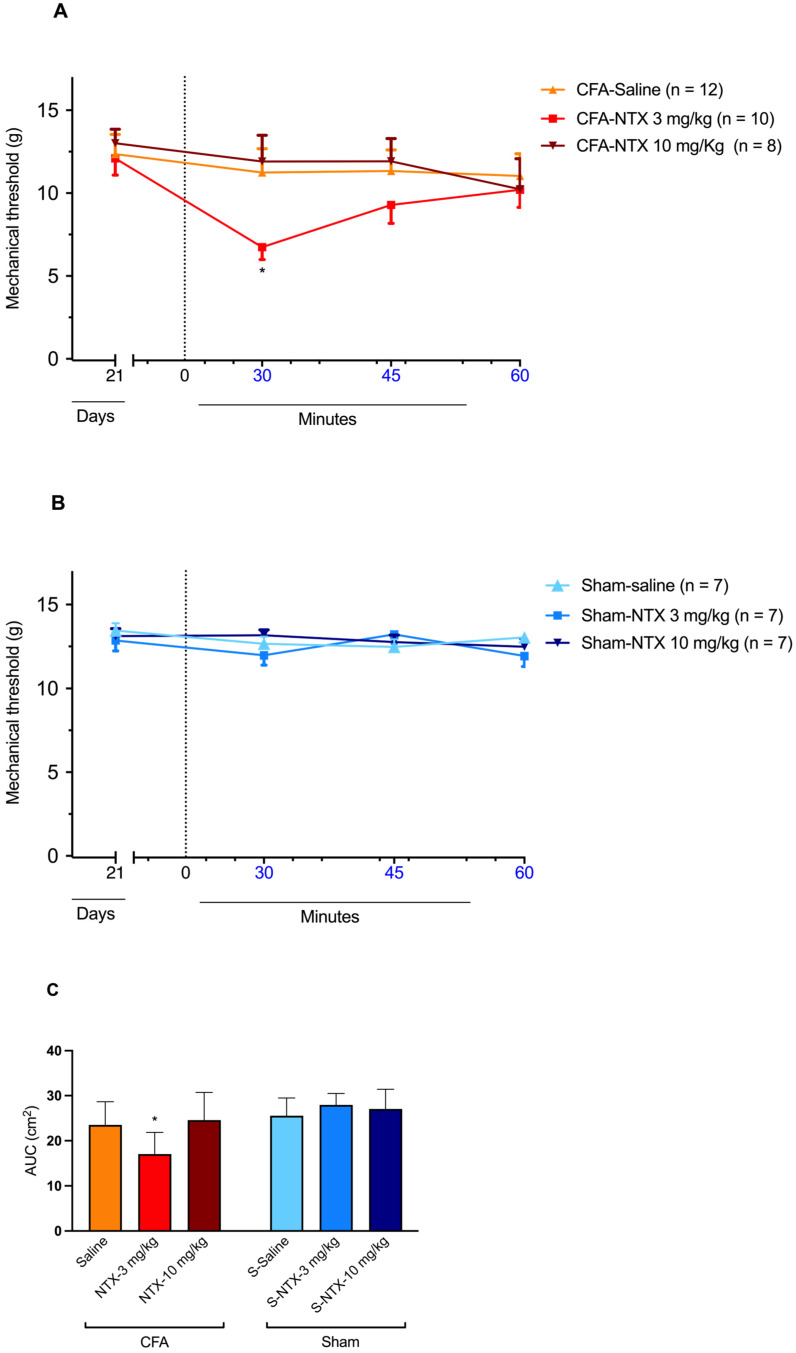
NTX-evoked reinstatement of mechanical allodynia in CFA-21d male Wistar rats. (**A**) The graph shows the time course of mechanical allodynia after subcutaneous administration of 3 mg/kg NTX (*n* = 12), 10 mg/kg NTX (*n* = 8), or saline (*n* = 10) to the CFA-injured rats on day 21 after CFA injection (CFA-21d rats; remission phase). * *p* < 0.05. Each point represents mean ± SEM. (**B**) The graph shows the time course of mechanical allodynia after subcutaneous administration of 3 mg/kg NTX (*n* = 7), 10 mg/kg NTX (*n* = 7), or saline (*n* = 7) to the sham-injured rats on day 21 after saline injection (sham-21d rats). Each point represents mean ± SEM. (**C**) The graph shows the AUC of data from graphs (**A**,**B**) for the section between 30 and 45 min post-administration of 3 mg/kg NTX, 10 mg/kg NTX or saline. * *p* < 0.05 (for 3 mg/kg NTX versus saline and 10 mg/kg NTX in CFA-21d rats). Each column represents mean ± SEM. See main text for abbreviations.

The subcutaneous injection of 3 mg/kg of NTX at CFA-21d rats resulted in a transient decrease in the mechanical threshold, indicative of the reinstatement of mechanical allodynia at levels similar to those recorded on D3 after CFA injection [two-way ANOVA, time F(3,81) = 3.100 * *p* < 0.05, treatment F(2,27) = 2.266 *p* = 0.12, and interaction F(6,81) = 1.432 *p* = 0.21] (Figure 2A). The effect was transient, as, after its detection at 30 min post-NTX administration, it gradually relented, returning to pre-NTX values by the end of the 60 min observation period. However, when administered at the higher dose of 10 mg/kg, NTX did not elicit any significant changes in the mechanical withdrawal threshold compared to the pre-NTX values during the monitoring period. Similarly, no significant effect on the mechanical withdrawal threshold was observed in CFA-21d rats following saline administration.

In the sham-21d rats, none of the three treatments produced significant changes in the mechanical threshold at any time post-administration [two-way ANOVA, time F(3,54) = 2.191 *p* = 0.09, treatment F(2,18) = 0.3533 *p* = 0.71, and interaction F(6,54) = 2.029 *p* = 0.08] (Figure 2B).

The different influence of the three types of treatments (NTX at 3 mg/kg, NTX at 10 mg/kg, saline) on the mechanical withdrawal threshold of CFA-21d rats was confirmed by the area under the curve (AUC) analysis. A significant decrease in NTX at 3 mg/kg was observed between 30 and 45 min post-treatment [one-way ANOVA, F(5,45) = 6.881 * *p* < 0.001] (Figure 2C). On the other hand, the same analysis confirmed the lack of influence of the three treatments on the mechanical withdrawal threshold of the sham-21d rats.

### 2.3. Effect of NTX Administration on the Behavioral Traits of CFA-21d Male Wistar Rats

**Figure 3:**
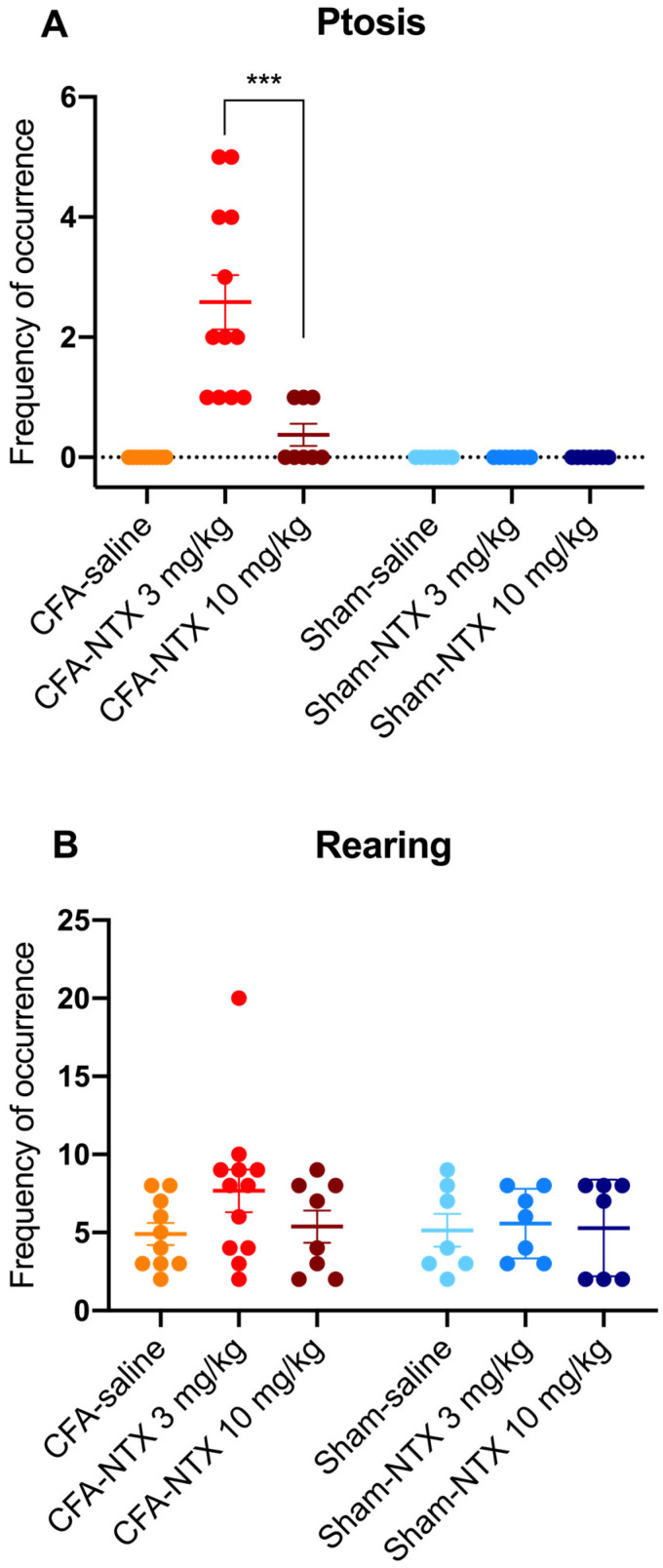
NTX-evoked opioid withdrawal behaviors in CFA-21d male Wistar rats. The column-scatter graphs show the frequency of occurrence of withdrawal-associated behaviors (number of behavior episodes counted over the 60 min observation period) after administration of 3 mg/kg NTX, 10 mg/kg NTX, or saline to CFA-injured rats on day 21 post-CFA intraplantar injection (remission phase; *n* = 12, *n* = 8 and *n* = 10, respectively) or to sham-injured rats on day 21 post-saline intraplantar injection (*n* = 7, *n* = 7 and *n* = 7, respectively). (**A**) Ptosis, (**B**) rearing. Individual points represent data from every animal, horizontal lines depict the mean, and vertical bars depict the SEM. *** *p* < 0.001 (3 mg/kg NTX versus 10 mg/kg NTX in CFA-21d rats). See main text for abbreviations.

Ptosis was not observed in any of the sham-21d rats (whatever treatment they received), nor in the CFA-21d rats treated with saline. Conversely, subcutaneous injection of NTX induced the appearance of this behavioral sign in the CFA-21d rats, though with differences between the two doses [one-way ANOVA, F(5,45) = 18.30 *** *p* < 0.001] (Figure 3A). Notably, all of the 12 CFA-21d rats treated with the lowest dose of NTX (3 mg/kg) exhibited ptosis during the 60 min observation period (prevalence of 100%), with a mean frequency of occurrence of 2.50 (±0.42) episodes/animal. In comparison, the group treated with the highest dose of NTX (10 mg/kg) showed significantly lower prevalence and frequency of ptosis. Indeed, ptosis was observed in only three out of eight animals (prevalence of 37.5%; Fisher’s exact test, ** *p* < 0.01), with just one episode being counted for each of them during the specified testing period (leading to a mean of 0.38 ± 0.18 episodes/animal; Sidak’s post hoc test, *** *p* < 0.001). Statistically, the prevalence of ptosis in the animals receiving 10 mg/kg of NTX was higher than in the saline-treated group (Fisher’s exact test, * *p* < 0.05).

Teeth chattering also was not observed in the sham-21d rats (independently of the treatment they received), nor in the saline-treated CFA-21d rats. In the group of CFA-21d rats treated with NTX at 3 mg/kg, this behavior occurred only in two out of twelve (prevalence of 16.7%), with just one episode per animal. This prevalence was not different on a statistical basis from the saline-treated group (Fisher’s exact test, *p* = 0.4831). Teeth chatters were not observed in any CFA-21d rats after administering the higher NTX dose (10 mg/kg).

Rearing activity was observed in all of the sham-21d and CFA-21d rats during the 60 min of observation, independently of the type of treatment received (prevalence of 100%) (Figure 3B). The mean frequency of occurrence of this behavior was numerically higher in the group of CFA-21d rats treated with the lowest dose of NTX (3 mg/kg) (7.64 ± 1.21 episodes/animal) in comparison with the other two groups of CFA-21d rats (saline: 4.92 ± 0.73 episodes/animal; 10 mg/kg NTX: 5.38 ± 1.03 episodes/animal), as well as in comparison with the three groups of sham-21d rats (saline: 5.14 ± 1.06 episodes/animal; 3 mg/kg NTX: 5.57 ± 0.84 episodes/animal; 10 mg/kg NTX: 5.29 ± 1.17 episodes/animal), but the difference was found to be not statistically significant [one-way ANOVA, F(5,45) = 1.049 *p* = 0.4009].

Wet dog shakes and paw tremors were never observed in any experimental group.

## 3. Discussion

Although similar in many aspects, mice and rats are not identical but present some fundamental differences in several aspects, including pain biology, with particular regard to the mechanisms underlying chronic pain [16] and addictive behaviors [15]. Such mouse–rat differences can be anatomical, physiological, biochemical, and/or pharmacological in their nature [16,22] and should be considered carefully when choosing a rodent model system for addressing specific research questions [15]. Indeed, the more advised the choice between the two rodent species, the higher the translational predictability of the preclinical research outcomes [15,16,23]. Moreover, in the pain drug discovery and development setting, it is well established that the risk of failure in advancing any compound to the clinic would likely be lower with a compound that is robustly efficacious in more in vivo preclinical pain models and would be even lower if efficacy was demonstrated in both rat and mouse models [6].

Accordingly, there is a valid reason to replicate the findings obtained in one rodent species in the other to identify possible interspecies differences [15]. In this view, further to previous observations made in a mouse CFA model [12,14], the present study investigated whether a rat CFA model is also characterized by the occurrence of a state of endogenous opioid dependence associated with a MOR_CA_-dependent latent pain sensitization.

To the best of our knowledge, this is the first study reporting the successful generation of the latent sensitization model of chronic inflammatory pain in Wistar rats. 

All previous studies on the rat version of this model had been conducted using the Sprague–Dawley rats [9,18,21]. Looking at pain sensitivity, the time course of the specific response of our Wistar rats to the triggering injury produced by the intraplantar injection of CFA was similar to that described for Sprague–Dawley rats by Marvizon et al. (2015), with a phase of hyperreactivity (mechanical allodynia) persisting for about 14 days, followed by complete remission by day 21 [9]. However, the magnitude of the nociceptive response to the inflammatory injury was overall smaller in the Wistar rats of the present study compared with that recorded previously in Sprague–Dawley rats by other authors [9,18,21], who found the mechanical withdrawal threshold to be reduced by about 80%, 70%, and 60% on days three, seven, and fourteen, respectively, post-CFA injection. Considering that the amount of CFA administered to the rats was the same in those studies and the present one, it seems likely that differences exist between the two rat strains concerning their reactivity to the inflammatory injury and/or sensitivity to pain [16]. Moreover, there is evidence that the relative contributions of lumbar spinal nerves to the composition of sensory fibers of the sciatic nerve differ significantly between rat strains, and this has been proposed to, in part, account for the inter-strain variability in the degree of neuropathic pain expressed after nerve injury in the spinal nerve ligation model [22]. The possibility that this or other unknown strain-related differences could also have implications for the behavioral pain outcomes of an inflammatory injury to the distal limb cannot be ruled out.

In the present study, the inflammatory nature of the pain hyperreactivity shown by Wistar rats in response to intraplantar injection of CFA was indirectly confirmed by the parallel development of paw edema. The time course of the development of the swelling component of the CFA-induced inflammation in our rats also was consistent with data from previous studies in both Wistar and Sprague–Dawley rats [5,8], reporting the presence of maximal paw edema starting from 24 h after the CFA injection, with at least seven days of persistence. It is worth mentioning that, in our study, a significant though minimal edema response to the proinflammatory agent could still be appreciated on day 21, namely in the absence of pain signs. Similar observations were made by other authors in CFA-injured Wistar rats [24], where paw edema was still present and pronounced on days 21 and 28 after the CFA injection, as well as in the CFA-injured mice (C57BL/6 strain) [12], where paw edema took about seven weeks to disappear completely. Therefore, our finding seems to confirm that edema and pain hyperreactivity associated with inflammation are independent responses [8] to be considered separately when using the rodent CFA model in the preclinical evaluation of the efficacy of novel anti-inflammatory and analgesic drugs [5]. At any rate, it must be acknowledged that in the chronic inflammation induced by CFA, an increased paw thickness may reflect not only edema but also the presence of an extensive inflammatory cell infiltration of the tissue that other studies have documented [24,25].

The fact that, in the CFA-injured Wistar rats used in the present study, the resolution of pain characterizing the remission phase of the model was not indicative of the extinction of pain sensitization but instead of the tonic suppression of this maladaptive state by the signaling of a population of constitutively active MORs was confirmed by the finding that NTX administration to the CFA-21d rats at the dose of 3 mg/kg reinstated mechanical allodynia. In addition, the finding that the sham-injured 21d rats did not develop mechanical allodynia in response to the same dose of NTX proved that the antinociceptive effect of NTX detected in the CFA-injured rats was dependent on the presence of sensitization. In agreement with observations made by Marvizon et al. (2015) in CFA-injured Sprague–Dawley rats [9], the time course of the antinociceptive effect of 3 mg/kg NTX in our CFA-injured Wistar rats (which was maximal at 30 min post-injection and had entirely disappeared by the end of the 60 min observation period) was consistent with the plasma elimination half-life of the drug in this rat strain, which has been reported to be of about 1 h [26]. The value of this pharmacokinetic (PK) parameter has been determined after the intravenous administration of a single bolus dose of 2.2 mg/kg of NTX. However, a PK study performed in man [27] showed that the PK profiles of NTX after intravenous and subcutaneous administration are similar, with the latter being characterized by fast absorption into the systemic circulation.

It is worth noting that in the abovementioned study by Marvizon et al. (2015), the reinstatement of mechanical allodynia was observed after subcutaneous administration of a lower dose of NTX (1 mg/kg) to the CFA-21d rats and caused a greater and more persistent decrease in the paw mechanical withdrawal threshold (significantly reduced by 60% and 30% at 30 and 60 min, respectively, after NTX administration) than the decrease recorded in the present study with a three-fold higher dose [9]. Again, this inconsistency may depend on the different pain sensitivity of the two different rat strains used in the present and previously published study [9] (Wistar and Sprague–Dawley, respectively) [16]. The possibility also exists that the inconsistent magnitude of the pain response of the two rat strains to the two doses of NTX reflected interstrain differences in the PK profile of this drug. Indeed, the hepatocyte metabolic profiles of various drugs have been shown to differ between these two rat strains [28], and the hepatobiliary system is believed to be a major route of elimination of NTX in rats [26].

There is, however, a further possible explanation for the highlighted inconsistency between the magnitude of the antinociceptive effect elicited by 1 mg/kg and 3 mg/kg of NTX in the rats of the referenced study [9] and ours, which can be proposed by also considering that the highest dose of NTX tested in our study (10 mg/kg) proved utterly unable to reinstate mechanical allodynia during the remission phase. Taken together, these observations suggest that the dose–effect relationship of NTX in rats (or, at least, in the Wistar rat strain) has an atypical, bell-shaped pattern that the present study reports for the first time. Further examples of a bell-shaped dose–effect relationship in the exertion of NTX activity can be found in the literature, as it was shown to have a potentiating effect on the activity of antiretroviral drugs on human lymphocytes [29]. However, our finding in the CFA-injured Wistar rats contrasts with previous observations in CFA-injured C57BL/6 mice [12], where NTX was found to induce pain reinstatement in a dose-dependent manner (i.e., yielding a classical inhibitory sigmoid dose–response curve), with the doses of 3 and 10 mg/kg being equally effective at eliciting the maximal possible effect [12]. This, incidentally, accounts for the fact that the last two doses were selected for the present study, with the expectation that they would have elicited similar maximal effects in rats.

The occurrence of bell-shaped concentration–response curves is not unusual with ligands of G-protein coupled receptors, but much remains to be understood concerning the mechanisms underlying this complex phenomenon [30,31,32]. Therefore, we do not have a clear explanation for the difference that seems to exist between the mechanical threshold response patterns of rats and mice to NTX. However, this data suggests that extreme caution is required when interpreting differences between one strain of rat versus one strain of mouse as true species differences exist and need to be confirmed by a head-to-head comparison in the same experimental setting [16].

A first hypothesis regarding the mechanisms behind the atypical dose–effect relationship of NTX in rats described in the present study can be formulated considering that high receptor expression levels have been reported to be a key factor in the detection of bell-shaped responses [31]. More specifically, in biological tissues expressing high levels of receptors, an agonist may induce its specific receptor to couple to different G-protein subtypes in a concentration-dependent manner so that the response of the same receptor population to low agonist concentrations differs from that to high agonist concentrations because of the receptor coupling to a different downstream G-protein mediated signaling cascade. This switch in G-protein coupling would not occur in tissues expressing moderate or low levels of receptors. Moreover, for the inverse agonist of 5-HT1_A_ receptors pindolol, it has been reported that actual inverse agonism is exerted in the presence of low receptor expression levels, while agonism is exerted in the presence of high receptor densities [31]. Based on this knowledge, it can be speculated that the different pattern of mechanical threshold response elicited by NTX in CFA-injured rats and mice reflects interspecies differences in the expression levels of the constitutively active MORs in the CNS regions involved in pain modulation, with rats presumably expressing higher MOR levels than mice. There is a substantial genetic distance between rats and mice [15]. Although this seems not to considerably affect the amino acid sequences of their MOR proteins (which show 97% homology) [33], interspecies differences have been found to occur in the overall organization of rat and mouse MOR genes, particularly involving the identity and location of several transcription initiation sites within the DNA sequence [34]. These rat-mouse MOR genomic differences, and potentially others not yet explored [35], can likely lead to species specificity in regulating MOR gene expression and responsiveness to endogenous transcriptional regulatory factors. In this respect, it is interesting to note that peripheral inflammation has been proven to induce changes in the expression of the MOR gene in rodents [36]. In addition, two studies conducted separately in CFA-injured rats [37] and mice [38], have shown up-regulated and down-regulated MOR expression, respectively, in response to the inflammatory insult. Based on this knowledge, we can speculate that the rat-mouse differences in the expression levels of MORs hypothesized above may reflect, at least in part, species specificity in the influence exerted by the inflammatory insult on the MOR gene expression. If that is the case, then the MOR gene should be included among the many genes regulated by chronic pain states of both neuropathic and inflammatory origin for which considerable transcriptomic variation between rats and mice has already been documented [16,39].

Genetic differences specifically impacting the activity of opioid receptors have also been highlighted among rats, mice, and humans [40,41]. So, the possibility that the differently shaped patterns of the pain response of the CFA-injured rats and mice to NTX highlighted in the present study may reflect species-related differences in the MOR expression levels and in the MOR functionality consisting, for instance, in different levels of MOR constitutive activity, which can influence both the amplitude and direction of the response observed, cannot be ruled out [31].

Further explanations for the atypical (bell-shaped) versus typical (sigmoidal) dose–effect relationship shown by NTX in evoking pain reinstatement in the CFA-injured rats (present study) and mice [12], respectively, can be hypothesized if it is considered that NTX, besides acting as an inverse agonist with high affinity at MORs, also interacts with lower affinity with the other two classical opioid receptors of the κ- and δ- type (KORs and DORs) [42]. Sustained activation of KORs and DORs is involved (along with increased MOR constitutive activity) in the maintenance of pain sensitization during remission in both mice and rats with CFA-induced chronic inflammation [21]. Therefore, a contribution to the differences highlighted in the pain response of CFA-injured rats and mice to NTX may also come from species-related variations in the KOR and/or DOR genes, leading to different expression levels and/or functionality of the respective receptor proteins encoded. Some support for this hypothesis can be gathered from Walwyn et al. (2016) [21]. In that study, the administration of the DOR selective antagonist naltrindole produced a reinstatement of pain hypersensitivity that, in mice, was of comparable magnitude to that produced by NTX, while in rats, it was much smaller. On the contrary, the administration of a KOR selective antagonist resulted in a pain reinstatement magnitude that was similar to that evoked by NTX in rats but slightly smaller when compared to the magnitude observed in mice. This suggests that the relative contribution of KORs and DORs to the state of endogenous opioid analgesia may differ between the two rodent species, with the contribution of DORs being probably larger than that of KORs in mice and likely smaller than that of KORs in rats. 

Furthermore, there is evidence that the direction of the effects evoked by KOR antagonists depends on the modulation site, with KOR antagonism at the spinal cord leading to pain facilitation and KOR antagonism at the amygdala leading to pain inhibition [43]. In this view, a possible relative abundance of pronociceptive KORs in the rat amygdala of the rat may explain the bell-shaped dose–(anti-analgesic) effect relationship of NTX in this species in the light of the so-called “dual receptor theory” or theory of the “noncompetitive auto-inhibition”, previously proposed for the bell-shaped dose–response curve of buprenorphine [32,44]. More specifically, high concentrations of NTX (like those presumably achieved after the administration of the higher dose selected for the present study—10 mg/kg) would simultaneously block this second site of lower affinity, counteracting the antinociceptive effect produced by the action at the first receptor site of higher affinity (MORs).

Besides the hypotheses formulated so far, essentially based on the pharmacodynamic aspects of NTX interaction with the target receptors, other possible causative mechanisms for the atypical (bell-shaped) dose–response relationship displayed by NTX in rats can be thought to be perhaps related to the NTX PK profile, which will need to be proven by further work. For instance, rats can metabolize NTX to 6β-naltrexol [45,46], which is known to act as a neutral antagonist at MORs and can competitively prevent the inverse agonist NTX from producing its inhibitory effect on the constitutively active MORs in both CFA-injured rats [21] and mice [12]. Therefore, we can speculate that the bell-shaped dose–response relationship of NTX in rats might reflect competitive antagonism by this active metabolite, similar to one of the mechanisms proposed to account for the bell-shaped dose–response curve of buprenorphine [32]. However, 6β-naltrexol is not a major metabolite of NTX in rodents (differently from what is observed in humans) [26] and, to the best of our knowledge, differences between rats and mice in the formation of this metabolite have not yet been reported.

In the present study, the subcutaneous administration of 3 mg/kg of NTX to the CFA-21d rats also proved able to precipitate some of the behavioral symptoms of the endogenous opioid withdrawal that Corder et al. (2013) first described occurring in CFA-21d mice receiving the same NTX dose [12]. Notably, 3 mg/kg of NTX consistently and distinctly induced the appearance of ptosis in the treated CFA-injured rats but not in the sham-injured rats used as controls, and this suggests that rats too (at least rats of the Wistar strain) are likely to develop a condition of endogenous opioid dependence in association with the state of latent pain sensitization maintained by the endogenous activation of the opioid receptor system.

However, it is interesting to note that, in CFA-injured mice, multiple and robust behavioral changes have been reported to characterize the NTX-precipitated endogenous opioid withdrawal, including not only the abovementioned ptosis but also enhanced rearing and overall locomotion, as well as the presence of jumping activity, teeth chatters, wet dog shakes, and paw tremors [12,14]. Conversely, in the NTX-treated CFA-injured rats of our study, only ptosis was present. Some of these rats also showed teeth chattering, which we never observed in their saline-treated counterparts (nor in the sham-injured controls). However, the prevalence of this event was too low (2/12) to allow a cause–effect relationship with the administration of NTX (3 mg/kg) to be established on a statistical basis. Similarly, rearing behavior was only slightly and non-significantly increased by 3 mg/kg of NTX, nor was the occurrence of wet dog shakes and paw tremors ever observed.

Regarding possible NTX-induced alterations involving overall locomotion and jumping activity, no conclusions can be drawn because our observation chambers were unsuited for a reliable evaluation of these behaviors, which were not taken into account in our study. Moreover, more accurate information regarding the effect of NTX on rearing (and, possibly, information on other anxiety-sensitive related behaviors) could have probably been obtained by additionally subjecting the rats to an open field test [47], but this was not performed in our study.

Given these limitations, we cannot rule out that other behavioral alterations not detected in this study might be involved in the CFA-injured Wistar rats’ endogenous opioid withdrawal syndrome. Our present results provide enough evidence to support the claim that the notion that rats and mice diverge in how they experience withdrawal from opiates (and other drugs of abuse) [12,15,48] also applies to withdrawal from endogenous opioids.

Interestingly, the behavioral traits of endogenous opioid withdrawal depicted for CFA-injured Wistar rats in this study also seem to differ from those usually described in both Wistar and Sprague–Dawley strains of this rodent species for exogenous opioid withdrawal. Indeed, the latter condition is commonly associated with behaviors that, besides ptosis, also include (among others) increased rearing and distinct occurrence of teeth chatters and wet dog shakes [48,49,50,51]. A possible explanation for these differences is that the specific set of behavioral manifestations involved in a withdrawal syndrome may vary qualitatively and/or quantitatively not only among species (or strains) but also within the same species (and strain) depending on the nature and intensity of the withdrawal and the related state of dependence (e.g., withdrawal from exogenous opioids versus withdrawal from endogenous opioids; the specific type of opioid causing addiction; withdrawal precipitated by antagonist administration versus withdrawal precipitated by abrupt discontinuation of the drug of abuse) [19,47,48,52].

Another noteworthy finding of the present study is that also with respect to the ability to precipitate withdrawal-associated behaviors, particularly ptosis, the dose–effect relationship of NTX in our CFA-injured Wistar rats proved to be atypical (bell-shaped). Indeed, when NTX was administered to rats at the higher dose of 10 mg/kg, ptosis occurred with much lower prevalence and frequency than in rats treated with the lower NTX dose (3 mg/kg), as it was observed just in three of eight rats and just once in each of them. Moreover, none of the other investigated withdrawal behaviors (teeth chattering, wet dog shakes, paw tremors, altered rearing), nor any other behavioral symptoms that could be suggestive of withdrawal, were observed in this group under our study conditions. This pattern of response suggests that complexity in the interaction of the nonselective opioid receptor inverse agonist NTX with opioid receptor populations of the CNS of the rat (Wistar strain at least) also extends to the areas of the brain that are involved in opioid addiction [14]. However, much work is still needed to substantiate this hypothesis, like all the other hypotheses proposed herein.

It should also be kept in mind that the present study, like most of the mice and rat studies about latent pain sensitization and chronic pain in general, was performed using male animals. Sex, however, is known as one of the factors that may influence individual susceptibility to develop chronic pain and addiction in human beings [53,54], and sex-related differences in the neural mechanisms underlying pain and addiction have been documented to occur in rodents [5,55,56,57], with possible species- and strain-specific variations in their expression [16]. Therefore, our findings in male Wistar rats, like previous findings in male Sprague–Dawley rats [9,18,21], do not necessarily apply to female rats, and further studies comparing males and females are needed to better define the translational validity of the rat CFA model (generated with either Wistar or Sprague–Dawley strains) as a tool for investigating the aspects of chronic pain considered in the present study [58,59].

## 4. Materials and Methods

### 4.1. Animals

Male Wistar rats, weighing from 230 to 250 g, were housed in proper cages (three to four per cage) in a dedicated room at a controlled temperature of 20–22 °C and relative humidity of 45 ± 10%. Food and water were available ad libitum, and a 12 h light/dark cycle was set up.

The experimental procedures employed in this study complied with the National Institutes of Health Guide for the Care and Use of Laboratory Animals, within the technical guidelines for the production, care, and use of animals in the laboratory issued by SAGARPA Mexico (NOM-062 ZOO-1999) and ARRIVE guidelines [60].

### 4.2. Study Design

The rats were randomly selected to form different groups and used in two experiments. Each rat was only used once for experimentation.

In the first set of experiments, 24 rats were acclimated for 60 min and then tested for BL mechanical withdrawal threshold and paw thickness. Immediately after BL data recording (D0), 17 of these animals were administered CFA to their left hind paw. Sham-injured rats (*n* = 7) were injected with an equal volume of saline and used as controls. The development of mechanical allodynia and tissue edema in the two groups was then monitored on D3, D7, D14, and D21 post-injection.

In the second set of experiments, 30 rats received an intraplantar injection of CFA (CFA-injured), and 21 received an intraplantar injection of saline (sham-injured). On D21 post-injection, immediately after assessing mechanical allodynia, the CFA-injured rats (CFA-21d rats) were randomly assigned to three different groups and subcutaneously administered with NTX at the dose of 3 mg/kg (*n* = 12), NTX at the dose of 10 mg/kg (*n* = 8), and saline (*n* = 10), respectively. Similarly, the sham-injured rats (sham-21d rats) were randomly assigned to three different groups (of 7 animals each) and administered with the same treatments as above (i.e., 3 mg/kg of NTX, 10 mg/kg of NTX, and saline, respectively). Assessment of mechanical allodynia was then repeated 30, 45, and 60 min after treatment administration. Moreover, for the whole 60 min period after treatment administration, the occurrence of behaviors that could be indicative of opioid withdrawal was recorded.

### 4.3. Experimental Procedures

#### 4.3.1. Protocol to Induce the Priming Inflammatory Injury with CFA

Undiluted CFA (Millipore-Sigma, Burlington, VN, USA) consisting of a 1 mg/mL suspension of heat-killed and dried *Mycobacterium tuberculosis* cells in 85% paraffin oil, and 15% mannide monooleate, was injected in a volume of 50 μL into the ventral-medial surface of the left hind paw of the rats as described in previous studies [5,8,9].

#### 4.3.2. Opioid Receptor Inverse Agonist Preparation and Administration

NTX hydrochloride (Sigma-Aldrich, San Luis, MI, USA) was administered to the rats by subcutaneous route. Specifically, the drug was dissolved in 200 μL saline up to reach the desired concentration and injected by the mean of a 27 G needle into the skin of the back, above the lumbar region, of the unanesthetized rats.

#### 4.3.3. Measurement of the Paw Mechanical Withdrawal Threshold

The mechanical withdrawal threshold of the hind paw was measured as an indicator of pain-associated mechanical allodynia. Measurements were performed as previously described [5]. Briefly, after acclimating to a stainless-steel grid, the animals were tested for mechanical allodynia with the von Frey monofilaments (Stoelting Inc., Wood Dale, IL, USA) by stimulating the mid-plantar region of the left hind paw with an incremental series of eight monofilaments of logarithmic stiffness. The 50% withdrawal threshold was determined using the modified up-down Dixon’s method [61]. Specifically, a monofilament number 4.31, exerting 2.0 g of force, was applied perpendicular to the plantar skin, causing a slight bending. A rapid paw withdrawal within 6 s was recorded as a positive response, and in that case, a smaller filament was applied. In case of an initial negative response, a larger filament was applied.

#### 4.3.4. Measurement of the Paw Thickness

The thickness of the treated hind paws was measured as an indicator of tissue edema. Measurements were performed using a precision caliper as previously described [8].

#### 4.3.5. Behavioral Assessment of Endogenous Opioid Withdrawal

Immediately after NTX or saline administration, CFA-21d rats were examined for the emergence of some behaviors usually associated with opioid withdrawal in this rodent species [52]. To this purpose, continuous videos were recorded for a period of 60 min and analyzed by direct observation by the same blinded evaluator.

The withdrawal behaviors assessed included: ptosis (defined as squinting of the eyes), teeth chattering (defined as teeth grinding or rapidly opening- crossing of jaws), rearing (defined as leaving both forepaws from the testing floor, and upward stretching of the body), wet dog shakes (defined as a rapid and sudden rotation of the head, neck, and shoulders from one side to the other, analogous to the way a wet dog may shake to dry itself), and paw tremors (defined as one or both paws shaking laterally).

For quantitative evaluation, the total number of episodes of the investigated behavior observed in each animal over the specified time interval was counted, thereby determining the individual’s absolute frequency of behavior occurrence. When no episode of the investigated behavior was observed in an animal (absent behavior), the individual frequency of behavior occurrence was scored as “0”. The mean frequency value of the group was then derived from the so-obtained individual data.

For groups where the investigated behavior was not consistently observed in all animals, the prevalence of behavior occurrence (i.e., the percentage of animals in a group exhibiting the behavioral symptom) was also considered to evaluate withdrawal.

### 4.4. Data Analysis and Statistics

The mean and the standard error of the mean (SEM) for each set of experimental data were calculated. In addition, mechanical withdrawal threshold data collected from CFA-21d and sham-21d rats between 30 and 45 min post-NTX or saline administration were also expressed as the integrated AUC. The normality of datasets was assessed by the Shapiro–Wilk test, and the significance of the differences observed was checked by using, as appropriate, one-Way or two-Way ANOVA (examining the interaction of treatment and time), followed by Tukey’s post hoc test for multiple comparisons. Furthermore, and where applicable, the prevalence of the investigated withdrawal behaviors was compared between groups by Fisher’s exact test.

In all cases, the significance threshold was set at * *p* < 0.05. All statistical analyses were carried out by GraphPad Prism software v. 8 (GraphPad Software Inc., San Diego, CA, USA).

## 5. Conclusions

In conclusion, to our knowledge, this is the first study to describe the occurrence of a long-lasting endogenous opioid-masked pain sensitization in a Wistar rat model of CFA-induced chronic inflammation. Furthermore, the present study is the first to demonstrate that this condition is associated with the development of endogenous opioid dependence. This adds to previous findings on mechanisms of long-term opioid inhibition of pathological pain described in mice. However, the two rodent models seem to differ in some of their responses to the pharmacological inactivation of the endogenous opioid receptor system with NTX, conceivably due to species-related factors that will be further explored in future studies.

Overall, our findings may have important implications in pain research, as they allow us to better define the usefulness of the rat CFA model as an experimental tool to study the pathophysiological mechanisms of chronic pain and opioid addiction development. In addition, it allows assessing the preclinical efficacy of new classes of drugs that may be relevant to these conditions. More in-depth knowledge of the characteristics of this rodent model, also in relation to the rat strain used for its generation, as well as how it differs from the homologous model developed in mice, will likely improve the ability of scientists to select the most appropriate rodent model for their research, increasing the likelihood of success of preclinical-to-clinical translation.

## Data Availability

The data presented in this study are available on request from the corresponding authors.
